# The Dirt on Clean Eating: A Cross Sectional Analysis of Dietary Intake, Restrained Eating and Opinions about Clean Eating among Women

**DOI:** 10.3390/nu10091266

**Published:** 2018-09-08

**Authors:** Michelle Allen, Kacie M. Dickinson, Ivanka Prichard

**Affiliations:** 1 Nutrition and Dietetics, College of Nursing and Health Sciences, Flinders University, G.P.O. Box 2100, Adelaide, SA 5001, Australia; soulfulavocado@gmail.com; 2 Health and Exercise Sciences, College of Nursing and Health Sciences, Flinders University, Adelaide, South Australia, G.P.O. Box 2100, Adelaide, SA 5001, Australia; Ivanka.prichard@flinders.edu.au; 3 SHAPE Research Centre, Flinders University, G.P.O. Box 2100, Adelaide, SA 5001, Australia; 4 Flinders Centre for Innovation in Cancer, Flinders Drive, Bedford Park, SA 5042, Australia

**Keywords:** clean eating, women, social networking sites, internet, dietary restraint, dietary intake

## Abstract

Clean eating is understood in broad terms to be an approach to eating which promotes the exclusion of processed foods. Social media and websites which promote clean eating are becoming increasingly popular as sources of nutrition information. Currently, there is a lack of knowledge regarding women’s opinions about clean eating sites and their influence on eating behaviour. The aim of the present study was to investigate differences in dietary intake, dietary restraint and opinions about clean eating between women who had, and women who had never adhered to dietary advice from clean eating sites. Using a cross-sectional survey design, women (*n* = 762) ranging in age from 17–55 completed a self-report questionnaire on eating behaviour and beliefs about clean eating. Findings showed that 25.5% of the sample adhered to dietary advice from a clean eating site sometimes, often or very often. A significantly higher proportion of women who had adhered to dietary advice from clean eating sites met dietary guidelines for the consumption of fruit, meats and alternatives compared to women who had seldom or never adhered. Adherers also had significantly higher levels of restrained eating and were more positive about clean eating in general in comparison to those who seldom or never adhered. Results provide new information about exposure to clean eating sites and how they may influence women’s eating practices. These preliminary findings suggest additional studies are required to better understand the influence of clean eating sites, particularly with regard to whether the information on such sites are from reputable sources and to what degree their recommendations may be problematic for individuals with eating concerns.

## 1. Introduction

Research shows that women are increasingly turning to non-traditional media outlets (e.g., social networking sites (SNSs)) seeking information about food and diet [1,2,3,4]. SNSs can be defined as a group of online applications which promote the communication and exchange of user-generated content, such as Facebook and Instagram [5]. The Internet and SNSs are key sources of health and nutritional information for women; US data reports that 68% of adult women use SNSs and 61% have looked online for health information within the past year [6]. In Australia, 60% of women aged over 18 are using SNS at least once per day and 44% look online for health information regularly [7,8,9].

Eating disorders represent the third most common chronic illness and the second leading cause of psychological illness for young females in Australia [9]. Both dietary restraint and body dissatisfaction are risk factors for disordered eating [10], and women are up to ten times more likely to have poor body image than men [11]. The media, and more recently, social media, is one of the most common external factors implicated in the development of body dissatisfaction and disordered eating [9,12]. Due to the rise in popularity of using SNSs as a source of health and diet information, exploring their potential impact on dietary behaviour and on the risk factors for disordered eating symptomatology among women is important.

SNSs are saturated with sites promoting clean eating. While there is no consensus or definition, clean eating has been described as “Choosing foods that are natural and wholesome—particularly foods that are free of chemicals, additives and preservatives, and refined, processed ingredients” [13]. Clean eating is a major consumer trend, with 40% of young people aged 18–30 years in the UK reporting dietary practices consistent with clean eating, for example excluding food groups such as dairy [14]. In the USA, the consumer demand for clean eating is even driving changes in the food supply. For example, manufacturers are reformulating food products to modify or remove certain ingredients and additives in response to consumer demand for “cleaner” processed foods [15].

Clean eating sites include personal web pages, and social media platforms or blogs dedicated to sharing an individual’s diet and lifestyle choices and to encouraging followers of the blog or webpage with examples of “healthy living” [2,4]. Authors of clean eating sites are often featured as health-related “experts” or “wellness gurus” in popular media outlets (e.g., television, Facebook and Internet articles). Some promote diet and lifestyle advice that claim to change a person’s life or cure certain illnesses and diseases [16]. Many do so without qualifications. Attractive and captivating photos of the authors are frequently included in order to convey to readers what following their particular diet can do for them [1,17]. Of concern is the potential for harmful dietary advice to be circulated amongst vulnerable users, in particular those who may have an eating disorder or are at risk of developing one [18,19]. For example, a content analysis of healthy living blogs, Boepple and Thompson [4], demonstrated that many ‘healthy’ living blogs contain content that emphasises thin appearance ideals and unhealthy messages about food and nutrition. In addition, many blogs were authored by individuals who identified themselves as having had an eating disorder, body image concern or displaying dietary restraint practices in the past [4].

Dietary restraint entails obsessive effort to restrict and control calorie intake and food choices and has been linked to disordered eating [20,21]. Studies have previously demonstrated that females are negatively impacted by media images which portray body type and lifestyle ideas that are unrealistic [22,23], and social media has become a new medium for body comparison and body image disturbance [24,25]. For example, a study by Lynch [1] analysed the entries of 45 blogs created by young women belonging to a food blogging community and found widespread promotion of attitudes and behaviours associated with dietary restraint. While there may be positive aspects to clean eating sites when delivering evidence-based and balanced information, there is cause for concern when this is not the case. An example of this are sites or blogs purporting that certain food groups, such as dairy or grains, be entirely eliminated from the diet, which is not in line with evidence-based nutrition guidelines and can lead to nutritional deficiencies [26] and potentially increase risk for chronic disease long term.

Despite the potential for clean eating sites to impact women’s attitudes towards health as well as their eating behaviour, empirical research in this area is limited. To date, there are only two reports in the literature that have examined clean eating. Both report on the negative social implications clean eating may lead to, through restrictive dieting practices and exclusion of multiple food groups [27,28]. As restraint is a general risk factor for disordered eating, it is important to look at how clean eating is linked to established risk factors for disordered eating.

The aim of the present study was to determine whether there are differences between diet adequacy, dietary restraint and opinions about clean eating in women who have followed dietary advice from clean eating sites and those who have not. Due to many clean eating sites promoting certain food groups and excluding others, it was hypothesised that women who followed dietary advice from clean eating sites would (1) be less likely to meet Australian Guide to Healthy Eating (AGHE) guidelines for all food groups, (2) report greater dietary restraint, and (3) express more positive opinions about clean eating than women who had never or rarely follow dietary advice from a clean eating site.

## 2. Materials and Methods

### 2.1. Participants

**Figure 1:**
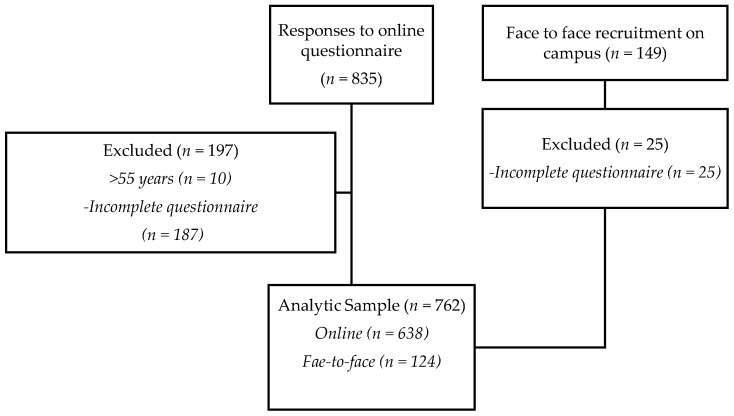
Participant flow for study recruitment face-to-face and online.

Participants were 762 women aged 17–55 years who completed a cross-sectional survey on “The Internet, Clean Eating, and Women’s Eating Behaviours”. Participants were recruited online via an advertisement on Flinders University’s research webpage and through professional clean eating, health and diet related social media pages which shared the link to the online study (*n* = 638) (Figure 1). Participants were also recruited face-to-face on Flinders University campus, South Australia) (*n* = 124) (83% return rate). Ethics approval was granted through Flinders University’s Social and Behavioural Research Ethics Committee, and return of the questionnaire was considered as informed consent. Inclusion criteria was female gender and age between >17 or <55 years. Participants were excluded if they were male or completed <50% of the survey (Figure 1).

### 2.2. Demographic Information and Social Media Use

All participants were asked to self-report their age, height and weight. Items measuring the frequency of use of social media platforms were adapted from Hay, Mond and Buttner [29] and included 10 items relating to Internet and social media use, the frequency with which respondents used the Internet during a typical week, and time spent on individual social media vehicles.

### 2.3. Self-Reported Adherence to Dietary Advice from Clean Eating Websites and Dietary Intake

To determine whether participants had ever adhered to advice from clean eating sites, they were asked to indicate how frequently they adhered to the dietary advice from one or more clean eating sites on a five-point Likert scale of ‘never’ to ‘very often’. Responses were then grouped such that participants who responded ‘never’ were considered to have never adhered to dietary advice, while participants who responded ‘seldom’ were considered to have rarely adhered to dietary advice, and the remaining responses ‘sometimes’, ‘often’ and ‘very often’ were coded as adherers to dietary advice. For the purpose of this study these three groups will be referred to as ‘non-adherers’, ‘seldom adherers’ and ‘adherers’ respectively.

Frequencies of core and non-core foods were assessed using ten short food questions that have been shown to reliably estimate food group consumption among Australian adults [30]. Participants were asked to indicate the number of serves per day they consumed for each of the following core food groups: fruit, vegetables, meat and alternatives (i.e., legumes, eggs, nuts and seeds), dairy, cereals and grains, and discretionary foods. Responses were dichotomised as either meeting or not meeting the AGHE recommended serves for each of the food groups per day for adult women [31]. This is consistent with population dietary surveys and other studies using online survey methods incorporating short questions to allow ranking of participants’ dietary intake (e.g., high/low) [32].

### 2.4. Dietary Restraint and Opinions about Clean Eating

Dietary restraint was assessed using the Restrained Eating sub-scale from the Dutch Eating Behaviour Questionnaire (DEBQ) [33]. The scale consists of 10 items which ask a range of questions regarding restriction of food and calorie restraint. Participants respond on a five-point ranging from 1 (never) to 5 (very often). A higher mean score indicated a greater level of restrained eating. Internal consistency for the present sample was high, *α* = 0.90.

In order to understand their opinions about clean eating, participants were asked to respond to an open-ended question at the end of the survey, “What is your opinion about clean eating?”

### 2.5. Data Analysis

Descriptive statistics were used to summarise demographic information. A one-way ANOVA test was used to examine differences in dietary restraint for non-adherers, seldom adherers and adherers, and between-group significance was tested with Scheffe’s post hoc pairwise method. Pearson’s chi-square tests were used to examine differences in the proportion of participants who had followed dietary advice from clean eating sites or not and whether they met AGHE guidelines, as well as their opinions about clean eating. A *p*-value of <0.05 was considered statistically significant. Data were analysed using IBM SPSS (Version 22, IBM Corp. Chicago IL USA). A minimum of 190 participants (95 in each group) were needed to demonstrate a medium effect at *p* < 0.01 when examining differences between groups. In looking at small effects for mean difference (dietary restraint) a total of 786 participants (322 in each group) were needed to demonstrate a small effect at *p* < 0.05 [34].

Preliminary and thematic analyses were conducted by the first author to examine emerging themes from the open-ended questions. The content from the questions was read intensively as a whole and then units of meaning were identified and given labels. These labels were then clustered together to incorporate coding of major themes [35], which were cross-checked with the other authors. Qualitative data was split into three major groups of overall opinions of clean eating: positive, negative and neutral. From this, three positive sub-themes (improves health; important concept; encourages healthy eating) and three negative sub-themes (food fad; potentially damaging; unrealistic) were derived. Responses which included multiple themes were coded into more than one sub-theme. This analysis was conducted blinded to participants’ quantitative data.

## 3. Results

### 3.1. Characteristics of the Sample

Participants were 762 women with a median age of 27 years (range = 17–55), weight of 65 kg (range = 38–153) and a BMI of 23.1 kg/m^2^ (range = 15.4–54.9) (Table 1). There were no significant differences between the adherers, seldom-adherers and non-adherers in terms of age, F(2, 758) = 1.764, *p* = 0.172, or BMI, F(2, 740) = 1.167, *p* = 0.312. There were also no significant differences between the three groups in terms of ethnicity, level of education, or the use of Facebook, Twitter or writing blogs (all χ^2^ < 15.08, *p* > 0.129). However, those that adhered to clean eating advice were significantly more likely to use Instagram and Pinterest, and read other people’s blogs (χ^2^ (10, *N* = 760) = 29.12, *p* < 0.001, χ^2^ (10, *N* = 760) = 31.90, *p* < 0.001, χ^2^ (10, *N* = 759) = 18.75, *p* < 0.05, respectively). Further demographic information of participants in each group is displayed in Table 1.

### 3.2. Dietary Intake

Table 2 shows the proportion of the overall sample, as well as a breakdown of adherers, seldom-adherers and non-adherers, who met the AGHE guidelines for each food group. Participants who adhered to clean eating advice were significantly more likely to meet AGHE guidelines for the consumption of fruit and meat and alternatives than participants who seldom-adhered or did not adhere to clean eating advice. There were no significant differences between groups for meeting adequate serves of vegetables, dairy, cereals, or discretionary foods.

### 3.3. Dietary Restraint

Overall the sample had a mean dietary restraint score of 2.63 (SD = 0.81) out of a total score of 5.0. Mean dietary restraint scores were significantly different between the groups overall, F(2, 729) = 26.934, *p* = 0.000 (adherers: M = 2.93, SD = 0.76; seldom-adherers: M = 2.71, SD = 0.75; and non-adherers: M = 2.42, SD = 0.80). Post hoc analysis showed that non-adherers had a significantly lower mean dietary restraint in comparison to both seldom-adherers and adherers (*p* = 0.000). Seldom-adherers also had significantly lower dietary restraint than adherers (*p* = 0.028).

### 3.4. Opinions about Clean Eating

Overall, 673 (88.3%) participants responded to the open-ended question regarding their overall opinion towards clean eating. Of those that responded, 39.7% (*n* = 267) held positive views of clean eating, 40.3% (*n* = 271) had a negative opinion, and 20.1% (*n* = 135) were neutral on the topic. When opinions of clean eating were examined in relation to whether participants adhered to dietary advice from clean eating sites, 63.9% (*n* = 108) had a positive opinion towards clean eating, 14.8% (*n* = 25) had a negative opinion, and 21.3% (*n* = 36) were neutral. Participants who had seldom adhered or never adhered were significantly less likely to be positive about clean eating, χ^2^ (4, *n* = 673) = 82.85, *p* < 0.001, 41.8% (*n* = 74) and 26.0% (*n* = 85), respectively, and were more likely to have a negative opinion about clean eating, 44.1% (*n* = 78) and 51.4% (*n* = 168) respectively.

Table 3 illustrates the sub-themes of positive and negative opinions towards clean eating. In looking at the sub-themes for positive opinions about clean eating, 21% (*n* = 139) of the entire cohort felt that clean eating improves health, 35% (*n* =2 32) thought it was an important concept and 46% (*n* = 310) believed it to encourage healthy eating. In contrast, 36% (*n* = 238) of the entire cohort thought clean eating was a food fad, 31% (*n* = 207) felt it was unrealistic to adhere to, and 30% (*n* = 203) believed clean eating to be potentially damaging. The most common sub-theme for adherers was that clean eating encourages healthy eating; 63% (*n* = 105) described this concept. With regards to non-adherers, 45% (*n* = 145) specified that clean eating was a food fad, and amongst seldom adherers, 36% (*n* = 63) thought it potentially damaging.

For participants that had a positive opinion towards clean eating, responses focused on three main themes, the promotion of healthy eating (e.g., “I think it’s a good thing. The basic idea of it is to focus on healthier foods—lots of vegies etc. which is healthy” 22 years, adherer), improved health (e.g., “I think clean eating is the answer to almost all health conditions and that a better quality of life can be attained by being aware and applying a good healthy regime of eating well”, 38 years, adherer), and clean eating being an important concept (e.g., “Clean eating is extremely important to ensure you get the most out of every day and feel the best you can” 21 years, adherer).

For participants who had a negative opinion towards clean eating, one of the main reasons cited was that it was a ‘food fad’ (e.g., “I think it is a bit of a fad and another buzz word around food. I think there needs to be better education about it for the general public” 33 years, non-adherer). The second main reason for participants having a negative opinion towards clean eating was the perception that it is potentially damaging (e.g., “It places too much emotion on foods—“bad” or “good”. This leads to negative feelings of self-worth when you eat un-clean food. Food is just food—it’s not good or bad” 28 years, non-adherer). Lastly, participants also stated a negative opinion about clean eating due to it being unrealistic to adhere to (e.g., “Clean eating is unrealistic and puts unnecessary pressure on people (particularly young woman) to eat in a ‘perfect way’. It creates a lot of guilt around eating food and doesn’t encourage a balanced lifestyle” 20 years, non-adherer). Some participants voiced conflicted opinions which were both positive and negative (e.g., “I think it’s a great principle but people can become too obsessed which leads to unhealthy guilt” 23 years, seldom adherer”; and “Difficult and time consuming but worth it” 26 years, seldom adherer).

## 4. Discussion

The aim of the present study was to explore the dietary intake, eating behaviour, and opinions of women who self-reported adherence to dietary advice from clean eating internet sites compared with those that reported seldom or no adherence. Results from the study suggest that self-reported increased usage of clean eating sites may impact upon women’s dietary behaviour, as well their general opinion about clean eating. In line with our hypothesis, women who adhered to dietary advice from clean eating sites reported significantly greater levels of dietary restraint and held more positive views about clean eating in general. Due to the potential exclusion of certain food groups when adhering to clean eating, it was also anticipated that women who had followed dietary advice from clean eating sites would be less likely to meet AGHE guidelines for different food groups. In contrast to this, findings indicate that women who adhere to dietary advice from clean eating sites are more likely to consume adequate amounts of fruit, as well as meat and alternatives. There were no other significant differences between groups for the other core foods. This suggests that for women who adhere to clean eating advice, fruits may be perceived as ‘safer’ foods. This is supported by prior work [1] which found that women who belonged to a food blogging community viewed fruits and vegetables as ‘safe’ foods, while high carbohydrate and discretionary foods were labelled as ‘naughty’, which was thought to be a result of the influence of group norms and attitudes within the blogging community. Additionally, Sztainer and French [36] found that women who adhered to certain diets for health reasons had higher fruit and vegetable consumption than women who did not follow any type of dietary practice. Future research could examine the motivation behind following clean eating advice to determine whether health-related reasons are a predictor of this type of eating behaviour.

Interestingly, while differences were found in the proportion of women eating adequate amounts of fruit, there were no significant differences between the proportion of women who had adequate intake of vegetables between women who adhered to clean eating advice and those who seldom or did not adhere. This may be due to the proportion of participants who actually met Australian guidelines overall. For example, 56% and 23% of the sample met the guidelines for fruit and vegetables respectively. This is similar (although slightly higher) to population data from the Australian Bureau of Statistics [37], which reported that while 53% of Australian adult women met the guidelines for fruit intake, only 9% met the guidelines for vegetable intake. As this study only looked at adequacy and not excess of whether guidelines were being met, it would be of interest to investigate whether following clean eating advice leads to overconsumption of fruit, particularly in the context of it not being associated with increased vegetable consumption.

It is also important to note that the entire sample had an inadequate intake of all core food groups. However, the proportion of women who consumed an adequate amount of all core foods groups except for grains was higher in our sample compared with women in a similar age group in Australia, suggesting our sample had a more nutritionally adequate diet overall [38]. This is concerning, especially for women of child bearing age who have increased requirements for particular nutrients including B-vitamins, folate, and calcium [37], of which the core food groups are important sources in the diet [39].

Results from the study suggest that women who report adhering to dietary advice from clean eating sites are more likely to exhibit dietary restraint. This is somewhat concerning, given that intense focus on quality of foods for clean eating on a daily basis may be unattainable by most people. Paired with dietary restraint, this may translate into implementing restrictive tendencies towards food, such as calorie counting or refraining from certain foods or food groups [40,41]. While dietary restraint is not a form of disordered eating, it can lead to more obsessive practices [42], with current research demonstrating that going on a diet is one of the strongest predictors of future incidence of developing an eating disorder [43,44]. The concept of clean eating can often encourage justification for following a certain diet, even if it is extreme in nature. The promulgation of clean eating messages on social media may act as a vehicle for obsession around food to take hold, particularly in those who have low health and nutrition literacy [16,45]. It is important to note that once grounded in young adults, dietary restraint practices can be difficult to alter [21], which is why health professionals should be concerned about the quality and source of dietary related information and advice promoted online. Unverified health advice like clean eating in an unregulated social media environment with almost 2.5 billion users [46] can be dangerous and costly if people believe and follow something that may not actually help them. In the most extreme cases, there are reports of serious health consequences as a result of micronutrient deficiencies, especially for children who have parents that follow and impose extreme healthy eating practices [47].

Qualitative findings exploring opinions about clean eating found that overall, those who reported adhering had a more positive attitude towards clean eating in comparison to the more negative opinions voiced by seldom and non-adherers. Paired with the fact that women who adhere were more likely to use Instagram and Pinterest, and read other people’s blogs, these findings suggest that, consistent with previous research [1,24,48], exposure to health and diet-related pages on social media may foster a positive viewpoint. Participants who held a positive view towards clean eating believed that it encouraged healthy eating, was associated with improved health, and was an important concept in general. In contrast, those with a negative opinion considered clean eating a food fad that was potentially damaging and unrealistic to adhere to. Overall, this suggests that while the ideal of healthy eating portrayed via clean eating is embraced as a good thing by some, other women are wary of information regarding nutrition and diet on these sites. It is possible that this may relate to whether the information presented on clean eating sites is evidence-based. This requires further exploration, as some previous research has demonstrated that women highly value healthy eating blogs written by a dietitian or other credible source [2], while in contrast to this, other research suggests that women place importance on short-term aesthetic benefits over long-term health when following dietary recommendations [44].

The findings of the present study should be considered in the context of some potential limitations. First, the study was cross-sectional in nature and therefore causal inferences cannot be made [49]. Second, we used a convenience sampling strategy. While females in the sample used for the current study were of similar weight and BMI compared with the wider Australian female population, it is possible that the results may not be representative of the general population of females. We conducted face-to-face recruitment as a way to address this; however, it is recognised that this is a limitation of the current study. Third, while we have examined the self-reported adherence to dietary advice from clean eating sites and its link to increased levels of dietary restraint, it is equally possible that those with high levels of dietary restraint seek out and adhere to information from clean eating sites, perhaps even as a way to justify their dietary restraint. It is possible that items such as dietary intake were subject to recall bias. Lastly, it was not within the scope of the research to determine whether participants viewed the clean eating sites as evidence-based or non-evidence-based, which is a limitation in terms of being able to make conclusions about the impact of these sites on women. We also did not ask participants to describe whether they followed a particular diet, for example, vegetarian or vegan, or whether they purposefully excluded particular foods from their diet, for example, whether they follow a wheat-free or dairy-free diet. Therefore, we cannot interpret which aspects of clean eating advice may be more or less harmful to one’s health.

Despite these limitations, the study provides an in-depth insight into the eating behaviour and opinions of women in relation to clean eating. In doing so, this study presents important new understanding and awareness of women’s perceptions of clean eating, as well as a rationale for exploring potential impact of exposure to clean eating sites and eating behaviour in future research.

Social networking use is positively related to body image concerns and disordered eating among both women and men [12]. As such, it is important that future research in this area includes the study of both genders to determine any differences in implications. As our findings have suggested a link between dietary restraint and clean eating, we need to understand the implications of this on risk of eating disorders, particularly among vulnerable groups like young adults. With over 2 billion active users on social media [46], the potential exposure and impacts of these nutrition trends on young people is unprecedented. The current study clearly demonstrates the need for further research on the potential damaging factors associated with following clean eating practices.

Findings from this study highlight the need for health professionals, including dietitians, to be informed about the diet messages presented online that the public is exposed to. In terms of future practice, there is a need for reliable health information regarding food and diet to be accessible on the Internet and social media, which is why dietitians and health professionals should be encouraged to become more involved in these spheres. This could be via writing their own blogs or partnering with existing food blog authors [26], as well as directing women to reputable web pages such as the Storehouse [50], which is a database of food blogs written by Accredited Practicing Dietitians and qualified nutritionists.

## 5. Conclusions 

In summary, self-reported exposure to clean eating sites is associated with differences in women’s eating behaviour and increased dietary restraint practices, as well as being linked to more positive opinions about clean eating. While findings from the present study show adherers to be more likely to meet the dietary guidelines for fruit and meats and alternatives in contrast to seldom and non-adherers, their higher level of dietary restraint raises the question of whether attempting to follow a ‘clean’ diet may lead to obsessive eating habits in certain women. While clean eating sites continue to be a popular medium for women as they increasingly turn to non-traditional media outlets for information, dietitians and health professionals need to demonstrate leadership in correcting potential misinformation, reducing the risk of problematic eating and upholding evidence-based and balanced eating habits in the online space to protect the public.

## Figures and Tables

**Table 1:** Participant characteristics stratified by self-reported adherence to dietary advice from clean eating sites.

	Whole Cohort	Adherers	Seldom-Adherers	Non-Adherers
	*n* = 762	*n* = 195	*n* = 199	*n* = 368
Age	27 (17/55) ^a^	25 (17/53)	28 (12/55)	27 (17/55)
Height (cm)	166 (143/186)	167 (150/182)	165 (145/179)	166 (143/186)
Weight (kg)	65 (38/153)	65 (40/153)	64 (40/149	65 (38/141)
Current BMI (kg/m^2^)	23.1 (15.4/54.9)	23.0 (16.4/54.9)	23.2 (17.3/54.7)	23.2 (15.4/48.4)
Postgraduate university degree *n* (%)	144 (19)	33 (17)	39 (20)	72 (20)
Undergraduate university degree *n* (%)	283 (37)	66 (34)	80 (40)	137 (37)
Caucasian *n* (%)	664 (87)	170 (87)	171 (86)	323 (88)
De facto relationship *n* (%)	169 (22)	47 (24)	37 (19)	85 (23)
Married *n* (%)	253 (33)	54 (28)	78 (39)	121 (33)
Social media use 5–10 h per week *n* %)	250 (33)	66 (34)	74 (37)	110 (30)
Social media use >10 h per week *n* (%)	297 (39)	72 (37)	67 (34)	158 (43)

^a^ Range (minimum and maximum) appear in parentheses following medians.

**Table 2:** Percentage comparison of whole cohort, as well as adherers, seldom-adherers and non-adherers, for meeting AGHE guidelines, as well discretionary food consumption.

Food Groups ^a^	Serves per Day ^b^	Number (Percentage of Respondents) Meeting Requirements					χ^2^ (df, *n*)	*p*-Value
		Entire Sample	Adherers	Seldom-Adherers	Non-Adherers	Females 19–50 y ^c^		
Fruit	2.0	425 (55.8)	123 (16.2)	113 (14.9)	189 (25.0)	(20)	7.04 (2, *n* = 757)	0.030 *
Vegetables	5.0	176 (23.1)	49 (6.4)	49 (6.4)	78 (10.2)	(4.2)	1.41 (2, *n* = 761)	0.493
Meat and alternatives	2.5	352 (46.2)	110 (14.4)	89 (11.7)	153 (20.1)	(5.3)	11.52 (2, *n* = 762)	0.003 *
Dairy	2.5	105 (13.8)	25 (3.3)	21 (2.8)	59 (7.8)	(6)	3.45 (2, *n* = 760)	0.178
Cereals and grains	6.0	27 (3.5)	3 (0.4)	6 (0.8)	18 (2.4)	(8.5)	4.44 (2, *n* = 761)	0.109
Discretionary foods	2.5	737 (96.7)	187 (24.6)	195 (25.6)	355 (46.6)	NA	1.16 (2, *n* = 761)	0.561
Discretionary foods < 1	<1.0	197 (25.9)	61 (8.0)	53 (7.0)	83 (10.9)	NA	5.31 (2, *n* = 761)	0.070

* *p* < 0.05. ^a^ All food groups data reported as *n* (%) out of the entire cohort. ^b^ Recommended number of serves per day as outlined by AGHE guidelines (NHMRC, 2016) for women aged 18–50. ^c^ Proportion of females aged 19–50 years meeting recommendations for number of serves from respective food groups from the Australia Health Survey 2011–2012 provided for comparison.

**Table 3:** Sub-themes regarding both positive and negative opinions towards clean eating (CE) for the entire cohort, split by adherers, seldom-adherers and non-adherers.

Sub-Themes about CE Opinions ^a^	Whole Cohort	Adherers	Seldom-Adherers	Non-Adherers	χ^2^ (1, *N* = 668)
	*n* = 668 ^b^	*n* = 167	*n* = 177	*n* = 324	
Positive themes					
Improves health *n* (%)	139 (21)	61 (37)	34 (19)	44 (14)	35.59 **
Important concept *n* (%)	232 (35)	85 (51)	62 (35)	85 (26)	29.58 **
Encourages healthy eating *n* (%)	310 (46)	105 (63)	73 (41)	132 (41)	24.29 **
Negative themes					
Food fad *n* (%)	238 (36)	39 (23)	54 (31)	145 (45)	24.43 **
Unrealistic *n* (%)	207 (31)	53 (32)	60 (34)	94 (29)	1.36
Potentially damaging *n* (%)	203 (30)	23 (14)	63 (36)	117 (36)	29.08 **

** *p* < 0.01. Adherers vs. non-adherers. ^a^ Participants were able to be categorised into more than one sub-theme based on their individual responses. ^b^ A total of 94 participants from the entire cohort of 762 chose not to respond to the open-ended question asking their overall opinion of CE.
